# Supplementary material for: A Global Screen for Assembly State Changes of the Mitotic Proteome by SEC-SWATH-MS
Source: Cell Syst. 2020 Feb 26;10(2):133–155.e6. doi: 10.1016/j.cels.2020.01.001 (PMC7042714; doi:10.1016/j.cels.2020.01.001)

Q9BW91 | NUDT9_HUMAN | NUDT9 NUDT10 PSEC0099 UNQ3012/PRO9771
Monomer MW [kDa]: 39.125 Monomer expected elution fraction: 48

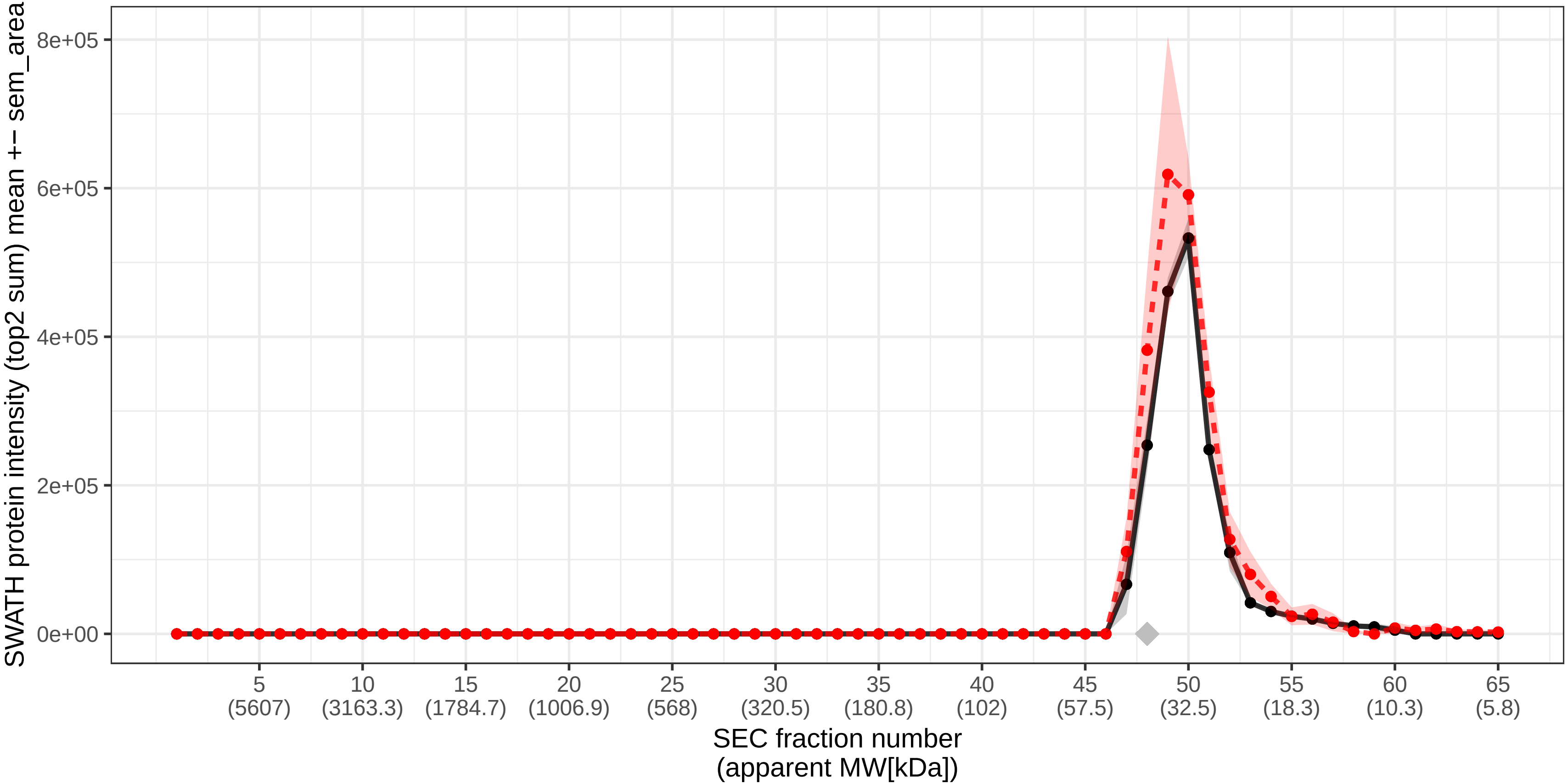

Supplement: Data S1. SEC-SWATH-MS Protein Chromatograms, Related to Figure 1 [file mmc6.zip › SECchrom_Q9BW91_NUDT9_HUMAN_NUDT9_NUDT10_PSEC0099_UNQ3012_P.pdf]
